# Supplementary material for: Do Hungarian multiple sclerosis care units fulfil international criteria?
Source: PLoS One. 2022 Mar 3;17(3):e0264328. doi: 10.1371/journal.pone.0264328 (PMC8893632; doi:10.1371/journal.pone.0264328)
Supplement: Attachment — Submitted filename: Response to Reviewers.docx [file pone.0264328.s001.docx]

**PLOS ONE Editorial Board, Emily Chenette (Editor-in-Chief), Michelle Dohm (Managing Editor), Marcello Moccia (Academic Editor), Anonymous Reviewers**

Dear Managing Editor,

I am writing to respond to each point raised by the Academic Editor and the Anonymous Reviewers regarding our manuscript entitled “Do Hungarian multiple sclerosis care units fulfil international criteria?”

We are grateful for the quick response and the helpful recommendations.

Our response to the recommendations is listed below:

Journal requirements:

1) PLOS ONE style requirements

PLOS ONE style requirements were thoroughly reviewed, and file names were altered according to the guideline. Filenames of the figures were consecutively changed from “Figure 1” to “Fig1.tif”, and filename of the manuscript was amended to “Manuscript”.

2) Acknowledgements
   The Acknowledgements Section was removed from the beginning of the manuscript. It only appears at the end of the manuscript after the Conclusions Section. Furthermore, the following statement was removed from the Acknowledgements Section:

“The current work was supported by GINOP 2.3.2-15-2016-00034, TUDFO/47138-1/2019-ITM and EFOP 3.6.3-VEKOP-16-2017-00009 project complementary scholarship for PhD students.”

At the same time, we would like to update our Funding Statement from:

“KZs was supported by University of Szeged Open Access Fund, grant number 5535. The Funder had no role in study design, data collection or analysis or preparation of the manuscript.”

to:

“KZs was supported by University of Szeged Open Access Fund, grant number 5535, and EFOP 3.6.3-VEKOP-16-2017-00009 project complementary scholarship for PhD students. VL was supported by GINOP 2.3.2-15-2016-00034, and TUDFO/47138-1/2019-ITM. The Funders had no role in study design, data collection or analysis or preparation of the manuscript.”

(This updated financial disclosure is also included in the updated version of the cover letter).

3) Ethics statement

The ethics statement in the original manuscript was already part of the Methods Section only, under the subheading “Ethical approval”. In the revision process to make the subheadings more transparent, consecutive numbering of subheadings throughout the manuscript were added. In case of not fulfilling the criterion “The ethics statement should only appear in the Method Section”, subheadings in the Method Section can be eliminated entirely, thus the ethics statement will be part of the Methods Section directly, as well as consecutive numbering of subheadings can be deleted throughout the manuscript as well.

4) Figure 1

Modified version of Figure 1 was created using one of the websites recommended by PLOS ONE. During the original review process at the end of November - beginning of December 2021 most websites recommended by PLOS ONE were under maintenance or could not be found, therefore a map strictly using Microsoft software was created and submitted, yet this version also encountered copyright issues. After receiving the e-mail that stated the modified version of the map still did not comply with the CC BY 4.0 license, the websites suggested by PLOS ONE were inspected again in January 2022. Fortunately, the second time around we were able to use one of the recommended sources. The process of creating the modified version is detailed below.

1. The apps.nationalmap.gov/viewer/ website was used to create the map.
2. Hungary was searched on the world map, and after zooming in a screenshot was taken, thereafter the screenshot was further edited.
3. Contrast and brightness levels were adjusted in Power Point so that only the country border remained distinct.
4. Approximate location of Hungarian multiple sclerosis care units was added (black dots and textboxes containing the name of each city were inserted).
5. The final product was saved as a picture in “.tiff” format.
5) Tables 1, 2 and 5

References to Tables 1, 2 and 5 were added to the appropriate paragraphs.

6) Reference list

The reference list was carefully reviewed, and the outdated/retracted reference was replaced with relevant, current reference.

(In the manuscript originally submitted, the outdated/retracted reference’s number was 22, however the replaced reference’s number is 23, because of the addition of a new reference, suggested by Reviewer #2)

Review comments made by the Reviewers:

Reviewer #1

1) Figure 2, lines 230-240

Figure 2 and legend of Figure 2 were changed according to the Reviewer’s observation, from:

“X indicates unfulfilled criterion, + indicates that all minimum and/or recommended criteria are met”

to:

“X indicates that criterion is met”

2) Table 6, lines 253-256

Table 6 and legend of Table 6 were altered pursuant to the Reviewer’s comment, from:

“✓ indicates that the disease modifying therapy is used in the care units, X with a grey background indicates that the disease modifying therapy is not used in the care unit, N/A with a grey background means that no data were supplied”

to:

“X indicates that the disease modifying therapy is used in the care units, cell with a grey background indicates that the disease modifying therapy is not used in the care unit, “N/A” means that no data were supplied.

3) Lines 265-270

Number of patients were added in each case, and percentages were placed into round brackets according to the Reviewer’s recommendation.

4) Lines 271-278

Number of patients were added in each case, and percentages were placed into round brackets in line with the Reviewer’s suggestion.

5) Discussion lines 285-403

The reference recommended by the Reviewer was added as a new paragraph to the Discussion Section:

“As previously mentioned, no surveys were conducted to assess whether or how MS care unit criteria are fulfilled in real-world practice. However, these criteria were carefully reviewed from a different aspect by a panel of neurologists from Latin America [20]. In Latin America diagnosis and treatment of MS was usually carried out by general neurologists, and care of people with MS was not perused in specialized centres, thus the panel’s objective was to create a realistic adaptation of the MS care unit recommendations considering regional differences regarding healthcare in Latin American countries. Cristiano et al acknowledged the importance of MSCUs to optimize MS care and reached consensus on what a care unit in Latin America should offer. However, they also concluded, that despite the desire to meet the personnel and instrumental requirements of an MSCU, it would be difficult to implement these expectations in certain regions of Latin America. Thus, they determined a standardized protocol for MRI assessments and recommended education of general neurologists to facilitate accurate diagnosis of MS. At the same time, they also proposed the idea of close collaboration between general neurologists and MSCUs, and similarly to our assessment they propounded the feasibility of reference care units.”

The reference recommended by the Reviewer was also added to the Reference Section:

“20.	Cristiano E, Abad P, Becker J, Carrá A, Correale JF, Fruns M, et al. Multiple clerosis care in Latin America: Consensus recommendations about its objectives and functioning implemetation. J Neurol. Sci. 2021 Oct 14;429:118072. https://doi.org/10.1016/j.jns.2021.118072”

Reviewer #2

1) The expression “immune modulatory therapy” was switched to “disease modifying therapy” in the Introduction Section, according to the Reviewer’s advice.
2) The term “MS patients” was altered to either “persons with MS”, “persons living with MS” or “people with MS”, “people living with MS” throughout the manuscript, in line with the Reviewer’s proposition.
3) Explanation of “why only data from Hungary were reported” suggested by the Reviewer, was added at the end of the Introduction Section’s Objectives Subheading:

“The present study is part of an international survey series conducted in DSNS member countries. Taking into account that such robust data collection is time and human resource demanding, data acquisition and analysis from DSNS member countries is in process. At the same time, considering the lack of similar assessments in the past, the present study can be considered as a pilot investigation aiming to collect data only from Hungarian MS care units, before presenting international outcomes.”

This rebuttal letter entitled “Response to Reviewers”, the updated version of the cover letter, entitled “Cover Letter”, the revised manuscript with track changes entitled “Revised Manuscript with Track Changes” will be included when submitting the revised version of the manuscript entitled “Manuscript”. Figure 1 originally portraying location of Hungarian care units was removed, and the rest of the figures were renumbered accordingly. Renamed versions of the figures entitled “Fig1.tiff”, “Fig2.tiff” will also be included when submitting the revised version of the manuscript.

Sincerely,

**Krisztina Bencsik MD, PhD (**Corresponding Author)

Department of Neurology, Faculty of General Medicine, Albert Szent-Györgyi Clinical Centre, University of Szeged, Szeged, Hungary.

6 Semmelweis street

Szeged, Hungary 6725

e-mail: bencsik.krisztina@med.u-szeged.hu

Tel: +36 62545351, Fax: +36 62545597

**Szeged, Hungary 6725**

**21.01.2022**
